# Mechanical, Viscoelastic, and Fracture Characteristics of Tea Waste and Boron Nitride Nanoplatelet-Reinforced Epoxy Composites

**DOI:** 10.1021/acsomega.6c00934

**Published:** 2026-05-14

**Authors:** Yasin Uslugil, Hüseyin Kaya, Mürsel Ekrem

**Affiliations:** † Department of Mechatronics Engineering, Faculty of Engineering and Nature Sciences, 218507 KTO Karatay University, Konya 42010, Turkey; ‡ Department of Machine and Metal Technologies, Technical Sciences Vocational School, 166263 Karamanoğlu Mehmetbey University, Karaman 70100, Turkey; § Department of Mechanical Engineering, 226846 Necmettin Erbakan University, Konya 42005, Turkey

## Abstract

This study presents the fabrication and characterization of sustainable hybrid epoxy nanocomposites reinforced with tea stem waste powder and 0.5 wt % boron nitride nanoplatelets (BNNPs). The tea stems were ball-milled to an average of 70 μm and added to the epoxy matrix in proportions ranging from 1 to 10 wt % relative to the epoxy resin. The mechanical performance was evaluated using Shore D hardness, tensile testing, and single-edge notch bending, while the thermal and viscoelastic behaviors were examined through dynamic mechanical analysis (DMA), thermogravimetric analysis (TGA), and differential scanning calorimetry (DSC) analyses. The hybrid composites exhibited significant enhancements compared with neat epoxy: Shore D hardness increased from 79.40 to above 97.00, tensile strength reached 63.09 MPa at 7.5 wt % tea stem, and fracture toughness improved from ∼1.70 to ∼2.10 MPa·m^1/2^. DMA revealed a clear glass transition at 94–97 °C, along with nearly double the storage modulus in the glassy region of the E-10 sample. The TGA results demonstrated increased thermal stability, with the residual char increasing to 9.58% for E-5. Although both 7.5 and 10 wt % tea stem contents yielded strong improvements, 7.5 wt % provided the best balance of strength, toughness, and filler efficiency. Overall, the findings show that untreated tea stem waste can be effectively combined with BNNPs to develop cost-efficient and environmentally friendly hybrid epoxy nanocomposites.

## Graphical abstract

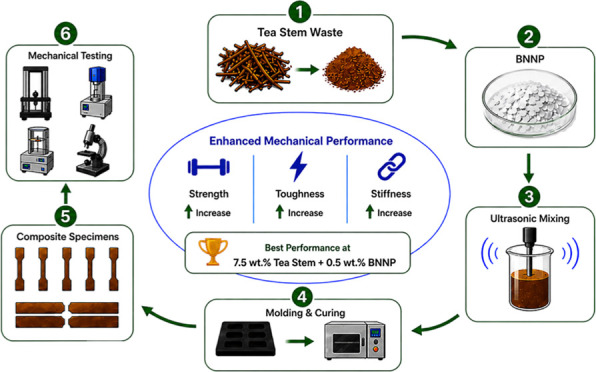

## 1 Introduction

Epoxy resins are thermosetting polymers widely used in modern industry due to their versatility and tunable properties. These materials are employed in automotive, marine, and civil engineering products, coating technologies, and electronic devices because of their characteristics.^1,2^ The excellent adhesion of epoxy to different parts makes it unique in its applications, including its tensile and compressive strength, dimensional stability, and remarkable chemical resistance.^3^ In addition, the ease of handling and ability to be modified toward a variety of applications using diverse curing agents is crucial.^4^

Despite the many advantages of epoxy resins, such as high-end-type materials for engineering, their application is restricted because composite materials still have deficiencies. Specifically, its inherent brittleness without fracture toughness and impact strength to resist dynamic loading and high stress renders it unstable.^5^ Furthermore, pure epoxy has low thermal conductivity, which is a significant barrier to its use as a material for heat-sensitive devices, such as electronic packaging and thermal interface materials.^6,7^ To overcome these deficiencies, multiple approaches have been used to modify epoxy matrices in combination with various fillers to optimize their mechanical, physical, and functional performances.^8,9^

As the performance requirements of such “green” composites become more demanding and the need to reduce the use of petroleum-derived products increases, interest in sustainable development is increasing.^10^ One of the approaches elaborated in this field is to cover natural fibers and agricultural waste byproducts with polymers. This is very promising because it is easily available and inexpensive.^11^ A wide range of lignocellulosic natural fibers, such as rice husks, wheat straw, kenaf, jute, sisal, and different agricultural residues, are used to improve the properties of polymer composites. Research in this area is rapidly developing.^12–14^ These natural fillers offer several advantages, including low density and renewable origin. Since lignocellulosic fibers are biodegradable in nature, their use as partial fillers in polymer composites can reduce reliance on fully synthetic materials. Furthermore, the use of these supplements can provide partial substitution of synthetic fillers, reducing the environmental impact and production costs.^15^ Despite these advantages, the use of natural fillers is challenging. Issues such as moisture absorption, dimensional instability, and weak interfacial bonding with hydrophobic polymer matrices often limit the degree of property enhancement that can be achieved.^16^ As a result, researchers are still working on surface treatments, chemical changes, and hybrid reinforcement methods to solve these problems and make the most agro-waste-derived materials in high-performance composites.^17–19^

Among various agricultural byproducts, tea stem waste is a promising lignocellulosic material for polymer reinforcement. Produced in large quantities during tea leaf processing, tea stem waste is abundant in tea-producing regions and is often underutilized or wasted.^20–22^ Tea stem waste is mainly composed of cellulose, hemicellulose, and lignin. These substances have been proven to provide stiffness, strength, and structural integrity to natural fibers. These factors make tea stem fibers a good filler for improving the mechanical properties of epoxy such as hardness, fracture toughness, and thermal resistance.^23^

Although natural fibers offer an economical production method, their contributions to polymer matrices are generally limited. To overcome this limited contribution and reach significant rates, nanomaterial reinforcement has been successfully applied and is a promising development in terms of its application in different materials.^24,25^ Carbon nanotubes, graphene and its derivatives, various metal oxide nanoparticles, and nanoclays are nanoscale composite additives that have been widely applied in epoxy and other polymer matrices.^26,27^ These nanomaterials provide efficient stress dissipation and can significantly improve the mechanical strength, stiffness, and thermal stability of the polymer matrices. From a mechanical point of view, nanofillers modify the crack propagation paths and create a barrier effect on the heat and mass transfer. They also facilitate more efficient load transfer between the matrix and the reinforcing phase.^28^ Furthermore, when nanofillers are combined with biobased or agrowaste reinforcements, synergistic effects can often be realized, where natural fillers provide sustainability and bulk reinforcement, while nanofillers impart advanced functionalities. Such hybrid approaches are increasingly being explored to design composites that simultaneously meet the performance requirements and environmental sustainability goals.^29,30^

In parallel with these developments, considerable attention has been directed toward thermally conductive yet mechanically compliant epoxy systems for multifunctional applications. Various filler strategies have been explored, including graphene and graphene nanoplatelets, carbon nanotubes, silver nanowires, metallic nanoparticles, and more recently MXenes and gallium-based liquid metal inclusions.^31–33^ In addition, three-dimensional interconnected hybrid networks based on metallic or carbon frameworks have been proposed to improve thermal transport while maintaining structural integrity.^34^ In polymer nanocomposites, the introduction of nanoscale fillers leads to a significant increase in the interfacial area between the filler and the polymer matrix. The polymer chains located near this interface form an interphase region whose dynamics differ from those of the bulk polymer and strongly influence the macroscopic properties of the material.^35^

There are many nanofillers in epoxy applications, and boron nitride nanoplatelets are representative of two-dimensional materials.^36–38^ BNNPs (boron nitride nanoplatelets) are often denoted as “white graphene” because of their hexagonal planar structure, similar to that of graphene.^39^ Boron nitride is an electrical insulator and is known for its relatively high thermal conductivity, chemical stability, and oxidation resistance. Because of these characteristics, BNNPs have attracted interest as reinforcing nanofillers in polymer composites and epoxy-based systems.^40,41^ Current studies show that the integration of BNNP into the epoxy matrix yields good results in terms of mechanical properties (such as fracture toughness and tensile strength), thermal stability, and dimensional consistency.^42^

Studies in which epoxy composites were reinforced with natural fibers alone or with nanoparticles showed remarkable improvements. In a study comparing cotton, sisal, coir, and wool fibers, the vacuum-assisted resin transfer molding (VARTM) method was used. It was observed that the composites produced with cotton fibers had a tensile strength of 52.81 MPa, while 15.34 MPa was measured for coir fibers. The correct fiber selection enables the achievement of a high tensile strength.^43^ In a study using Himalayan natural fibers, increased tensile strength was observed from 24.31 to 39.21 MPa with an increasing fiber ratio in composites prepared by the hand lay-up method.^44^ Lignocellulosic systems also improved significantly, with sisal, jute, and coir raising the tensile strength by nearly 200% over neat epoxy.^45^ Nanomodifications further advanced the performance: nanoclay-treated banana fiber composites showed 100% higher modulus and flexural strength,^46^ nanosilica at 6 wt % raised tensile and flexural strengths by ∼50% while reducing porosity,^47^ and CNT-reinforced bamboo/epoxy increased tensile strength from 27 to 33 MPa and flexural strength from 60 to 80 MPa, though impact strength decreased from 90 to 60 J/m.^48^

However, studies combining lignocellulosic agrowaste fillers with boron nitride nanoplatelets in epoxy systems remain very limited. In particular, the use of untreated tea stem waste as a reinforcement together with thermally functional nanofillers such as BNNPs has received little attention in the literature. The present study, therefore, explores a hybrid reinforcement strategy in which ball-milled tea stem waste particles are combined with a low loading of boron nitride nanoplatelets to enhance the performance of epoxy composites while maintaining the material sustainability and cost efficiency. Unlike many previously reported hybrid systems that rely on chemically treated fibers or carbon-based nanofillers, the present work focuses on untreated lignocellulosic tea stem waste combined with BNNPs as a cost-effective hybrid reinforcement for epoxy composites. In addition, the mechanical, fracture, viscoelastic, and thermal responses of the resulting hybrid composites are evaluated together in a systematic manner, giving a complete picture of the cooperative reinforcement mechanisms within this environmentally oriented epoxy composite system.

In this study, epoxy composites reinforced with tea stem waste and boron nitride nanoplatelets (BNNPs) are developed. Tea stem powders were prepared by ball milling, whereas BNNPs were used as received. The compositions included neat epoxy, epoxy with 1–10 wt percent of tea stem waste, and hybrid systems containing 0.5 wt % BNNPs. The composites were characterized through tensile testing, Shore D hardness, and fracture toughness measurements to evaluate their mechanical performance. The thermal behavior of the hybrid nanocomposites was investigated by differential scanning calorimetry (DSC) and thermogravimetric analysis (TGA). The influence of tea stem waste and boron nitride nanoplatelets on the temperature-dependent stiffness and damping characteristics (viscoelastic behavior) of the hybrid composites was also investigated using dynamic mechanical analysis (DMA). A schematic overview of the overall experimental workflow is provided in Supporting Information (Figure S3).

## 2 Materials and Methods

### 2.1 Material Preparation

Tea stem waste, collected as an agro-industrial byproduct, was initially cleaned with distilled water to remove adhering dust and surface contaminants, followed by drying in a hot-air oven at 70 °C for 6 h to eliminate residual moisture. Figure 1 shows the dried and fragmented stems.

**Figure 1:**
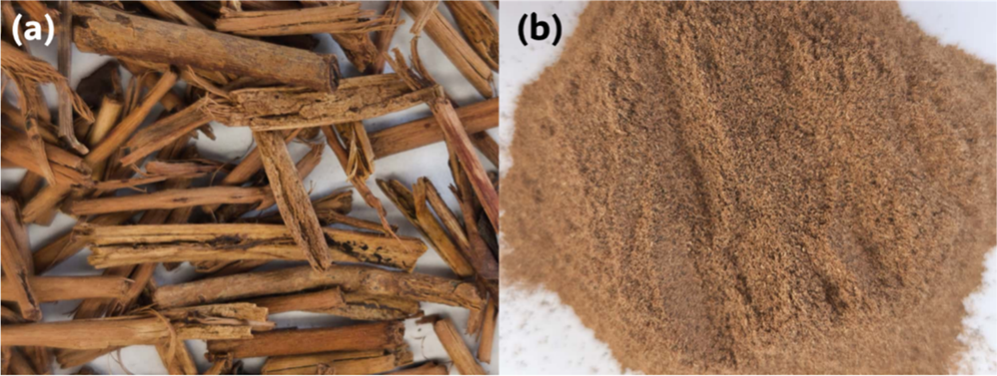
(a) Dried tea stem waste in its as-received form and (b) tea stem powder obtained after fragmentation using a high-speed blender.

The dried stems were mechanically fragmented using a high-speed blender (Retsch/GM200) for 5 min, which reduced the bulk size to coarse particulates, as shown in Figure 1a. Subsequently, the material was subjected to fine milling in a planetary ball mill (tungsten carbide vial, ball-to-powder ratio of 10:1, rotational speed of 300 rpm, milling time of 2 h), resulting in an average particle size of 70 μm (Figure 2). The measured distribution showed a d10 of 28–33 μm, d50 of 67–74 μm, and d90 of 118–132 μm, confirming that the powder consisted of relatively coarse, irregularly shaped particles with a broad size range. These values are consistent with the morphology observed in Figure 2 and reflect the inherent heterogeneity of untreated lignocellulosic fillers.

**Figure 2:**
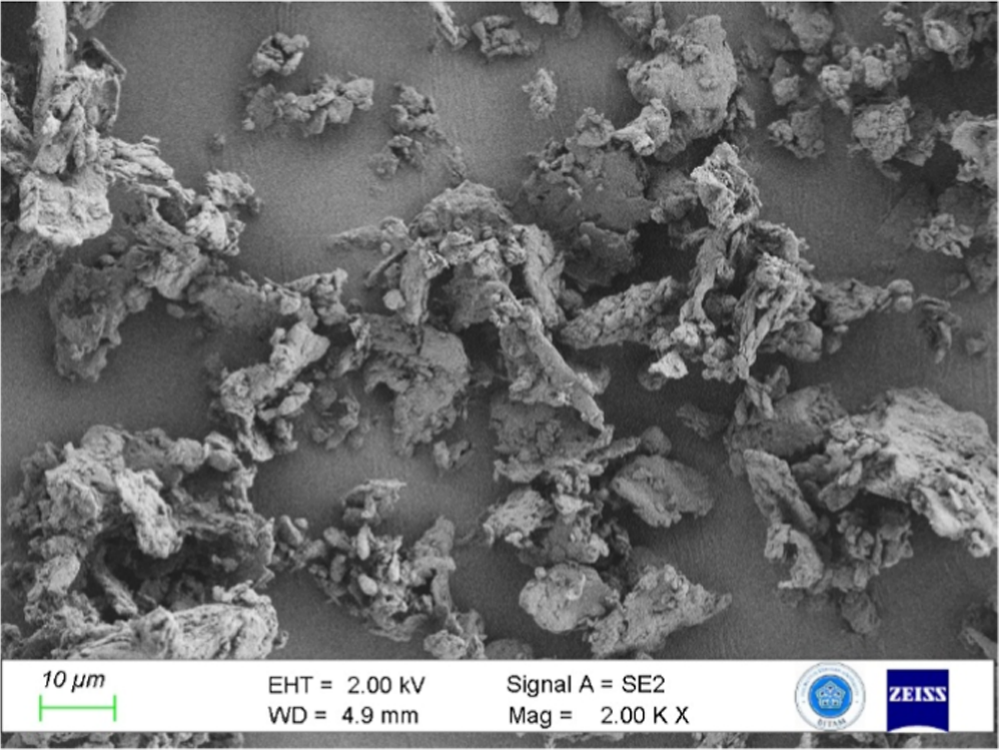
FESEM images of tea stem particles that were crushed and reaggregated after ball milling.

Commercial boron nitride nanoplatelets (BNNPs) with an average thickness of approximately 5 nm and a lateral size of about 58 nm were supplied by Eti Maden (Türkiye). The nanoplatelets exhibit a typical two-dimensional platelet morphology of hexagonal boron nitride nanosheets, as documented in previous studies.^49^ The epoxy matrix was prepared using the MGS LR285 epoxy resin and H285 curing agent supplied by Momentive Specialty Chemicals. According to the manufacturer’s technical data sheet, LR285 is a low-viscosity bisphenol A-based (DGEBA) laminating epoxy resin with a density of 1.18–1.23 g cm^–3^ and a viscosity of 600–900 mPa·s at 25 °C. The H285 curing agent is an amine-based hardener with a density of approximately 0.94–0.97 g cm^–3^ and a viscosity of 50–100 mPa·s at 25 °C. Predetermined weight fractions of the tea stem powder (1, 2.5, 5.0, 7.5, and 10.0 wt %) and BNNPs (0.5 wt %) were calculated relative to the weight of the neat epoxy resin before adding the curing agent. Tea stem powder and BNNP were dispersed with an ultrasonic probe and stirred mechanically afterward. Dispersion was performed by ultrasonication at 30% power at 2000 W for 15 min in an ice bath to improve dispersion and minimize agglomeration. The epoxy resin was then mixed using a mechanical stirrer at 1000 rpm for 10 min, followed by vacuum degassing to eliminate the trapped air bubbles. The curing agent was added at a 40:100 ratio relative to the epoxy resin in accordance with the manufacturer’s specifications, and the mixture was mechanically hand-stirred to ensure homogeneous blending. The compositions of the epoxy composites containing the waste tea stem powder and boron nitride nanoplatelets are listed in Table 1.

**Table 1:** Composition of Epoxy-Based Composites Reinforced with Powder from Waste Tea Stems and BNNPs

sample code	epoxy resin A [wt %]	hardener B [wt %]	waste tea stem powder [wt %]	BNNP [wt %]
E-0	71.43	28.57	0	0
E-1.0	70.67	28.27	0.71	0.35
E-2.5	69.69	27.88	1.74	0.35
E-5	68.49	27.40	3.42	0.34
E-7.5	67.33	26.93	5.05	0.34
E-10	66.23	26.49	6.62	0.33

### 2.2 Fabrication of Composites

The prepared epoxy, hardener, tea stem powder, and BNNP were thoroughly mixed according to their compositions, as listed in Table 1. The mixture was then poured into steel molds designed for the tensile (ASTM D638 type IV) and fracture toughness (ASTM D5045) specimens.

After casting, the molds were left to cure at 25 °C for 24 h, after which postcuring was applied at 80 °C for 15 h to ensure complete cross-linking of the epoxy. The specimens were then demolded, polished to remove surface irregularities, and stored under controlled conditions before mechanical and thermal testing. The molds used for specimen preparation and representative test samples are shown in Figures S1 and S2 in the Supporting Information.

### 2.3 Mechanical Characterization

Mechanical characterization of the epoxy-based composites was carried out using hardness, tensile, and fracture toughness measurements to evaluate the effect of the tea stem powder and BNNP on the structural performance of the material. Each test was performed using standardized specimen geometries and controlled loading conditions, as described in the following subsections.

#### 2.3.1 Hardness Test (Shore D)

Shore D hardness measurements were performed according to ASTM D2240 using a digital durometer. Five specimens were tested for each composition, and the hardness values were recorded at three different points on each sample. The results are reported as the mean hardness, together with the corresponding error bars.

#### 2.3.2 Tensile Test

Tensile tests were performed by using a Shimadzu AGS-X universal test device with a 10 kN load cell. The experiments were performed at a speed of 2 mm/min using specimens with dimensions expressed by type 4 specified in the ASTM D638 standard. Five samples were tested for each composite variant. The median of five specimens was determined as the representative specimen for that variant, and the force-elongation comparison curve was constructed from these specimens.

#### 2.3.3 Fracture Toughness Testing

A Shimadzu AGS-X universal testing machine was used for the fracture toughness and tensile tests, and the load capacity of the machine is 10 kN. The specimens were cast in metal molds in accordance with ASTM D5045, and the final end forms of the notches, which were first formed in the mold, were manually shaped. The specimens were 5 mm thick (*b*) and 20 mm wide (*w*). The initial machined notch was approximately 7.7 mm, and after introducing a sharp razor precrack, the total crack length (*a*) was measured and used in all fracture toughness calculations in accordance with ASTM D5045. The tests were conducted at a speed of 10 mm/min.

First, the temporary fracture toughness (*K*_Q_) is calculated for each specimen using eq 1, which includes the maximum load (*P*_Q_), the sample thickness (*B*), the width (*W*), and the corresponding geometric correction function *f*(*a*/*W*).

1 $$K_{Q}=\frac{P_{Q}}{B\sqrt{W}}f\left(\frac{a}{W}\right)$$

The geometric correction function *f*(*a*/*W*) was calculated using the shape function in eq 2, which accounts for the relative crack length (*a*/*W*). Together, eqs 1 and 2 provide the provisional stress intensity factor (*K*_Q_) required to assess plane-strain fracture behavior.

2 $$f\left(\frac{a}{W}\right)=\frac{3\sqrt{\frac{a}{W}}}{2{\left(1-\frac{a}{W}\right)}^{3/2}}\left[1.99-\frac{a}{W}\left(1-\frac{a}{W}\right)\left(2.15-3.93\frac{a}{W}+2.7{\left(\frac{a}{W}\right)}^{2}\right)\right]$$

The validity of the provisional stress intensity factor (*K*_Q_) was examined using the requirements specified in eqs 3–5: According to these criteria, both the specimen thickness (*B*) and remaining ligament (*W* – *a*) must satisfy the plane-strain size condition defined in eq 3, where the critical value depends on the ratio of *K*_Q_ to the material’s yield strength (σ_y_). Equation 4 provides the corresponding ligament requirement, ensuring that the uncracked section is sufficiently large to maintain small-scale yielding conditions. When both size requirements were fulfilled, as confirmed for all tested specimens, the provisional value (*K*_Q_) met the full validity conditions outlined in eq 5 and was therefore accepted as the true plane-strain fracture toughness (*K*_IC_).

3 $$B\geq 2.5{\left(\frac{K_{Q}}{\sigma_{y}}\right)}^{2}$$

4 $$W-a\geq 2.5{\left(\frac{K_{Q}}{\sigma_{y}}\right)}^{2}$$

5 $$K_{IC}=K_{Q}$$

The provisional stress intensity factor *K*_Q_ was calculated from the maximum load using the standard equation provided in ASTM D5045. The validity of each *K*_Q_ value was examined according to the fracture toughness criteria of the standard. The yield strength (σ_y_) of the epoxy and composite formulations was experimentally measured, and the obtained values showed sample-to-sample variations owing to the heterogeneous nature of the natural filler. To ensure a conservative and accurate evaluation, a σ_y_ interval of 65–75 MPa covering the full range of the measured values was used when applying the ASTM D5045 size requirements in eqs 3 and 4, respectively. For all specimens and for every σ_y_ value within this interval, both the thickness and remaining ligament conditions were fully satisfied.

Accordingly, all provisional *K*_Q_ values met the necessary plane-strain criteria and were, therefore, validly reported as *K*_IC_.

### 2.4 Dynamic Mechanical Analysis

The single cantilever beam model was used for the DMA, which was performed at a frequency of 1 Hz with a sinusoidal dynamic force with an amplitude of 1 N and an increasing temperature curve. The specimen is in the form of a rectangular prism, 25 mm long, 10 mm wide, and 3 mm thick. DMA was conducted at the BİTAM laboratories, which are the central laboratories of Necmettin Erbakan University, using a PerkinElmer 8000 DMA device.

The sample temperature, program temperature, dynamic and static force, in-phase and out-of-phase angles, stiffness, storage modulus (*E*′), loss modulus (*E*″), tan δ, and displacement-based quantities, such as dynamic strain and static strain, are among the many mechanical and viscoelastic parameters that the DMA instrument continuously recorded. The internal software of the system also automatically calculated other parameters, including viscosity-related values, complex modulus, shear storage modulus, shear loss modulus, and corrected total stiffness.

### 2.5 Fourier Transform Infrared Spectroscopy (FTIR)

Fourier transform infrared (FTIR) spectra were recorded using a Thermo Scientific Nicolet iS20 FT-IR spectrometer equipped with an ATR (attenuated total reflectance) accessory. The spectra were collected in the 4000–400 cm^–1^ wavenumber range with a spectral resolution of 4 cm^–1^. A background spectrum was acquired before each measurement, and ATR correction was applied using the instrument software. The spectra were baseline-corrected and normalized for comparison.

## 3 Results

### 3.1 Hardness Test

As illustrated in Figure 3, the incorporation of tea stem powder together with 0.5 wt % BNNP leads to a clear and progressive increase in Shore D hardness across the composite series. Neat epoxy (E-0) exhibited an average hardness of 79.40, representing the baseline performance of the unmodified matrix. When 1 wt % filler is introduced (E-1), the hardness rises substantially to 95.60, indicating that even a relatively low concentration is sufficient to increase the hardness and resistance to localized surface deformation of the epoxy.

**Figure 3:**
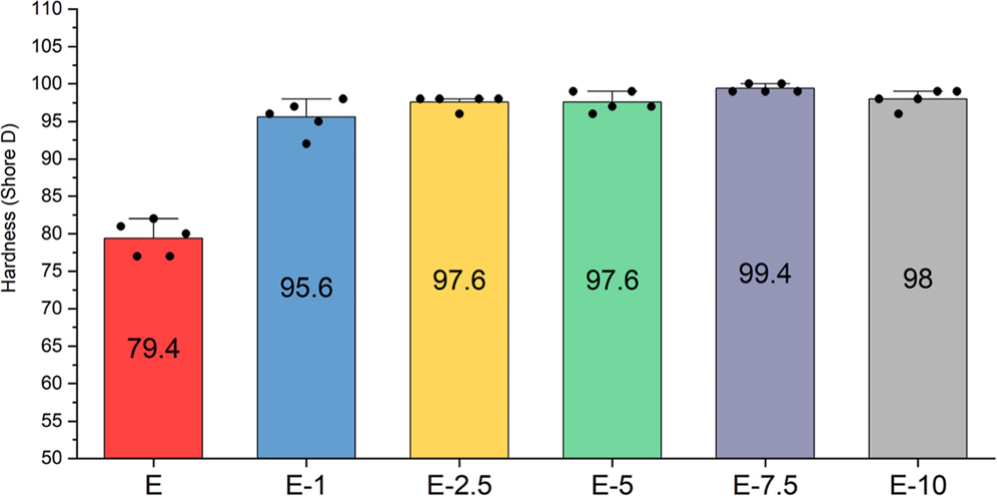
Shore D hardness values of the composites.

When the samples (E-2.5 and E-5) containing 2.5% and 5.0% tea stem powder by weight were examined, it was observed that both had an average hardness value of 97.60 Shore D. The highest values were observed for samples E-7.5 and E-10, although they were very close to those of each other. Accordingly, it is observed that the increasing amount contributes to the hardness, but it reaches an optimum value of approximately 7.5%, and no further improvement is achieved.

### 3.2 Tensile Testing

When the combinations of samples containing 0.5% BNNP with varying amounts of tea stem powder were examined, it was observed that increasing the amount of tea stems had a positive effect on the tensile strength of the samples. When 1% tea stem was added to a constant amount of BNNP (0.5%), an improvement of approximately 12% was achieved compared to pure epoxy, as shown in Figure 4. The 7.5% tea stem resulted in 63.09 MPa, and 10% tea stem resulted in 60.75 MPa. In this study, we analyzed the median samples and found that 7.5% yielded the highest results.

**Figure 4:**
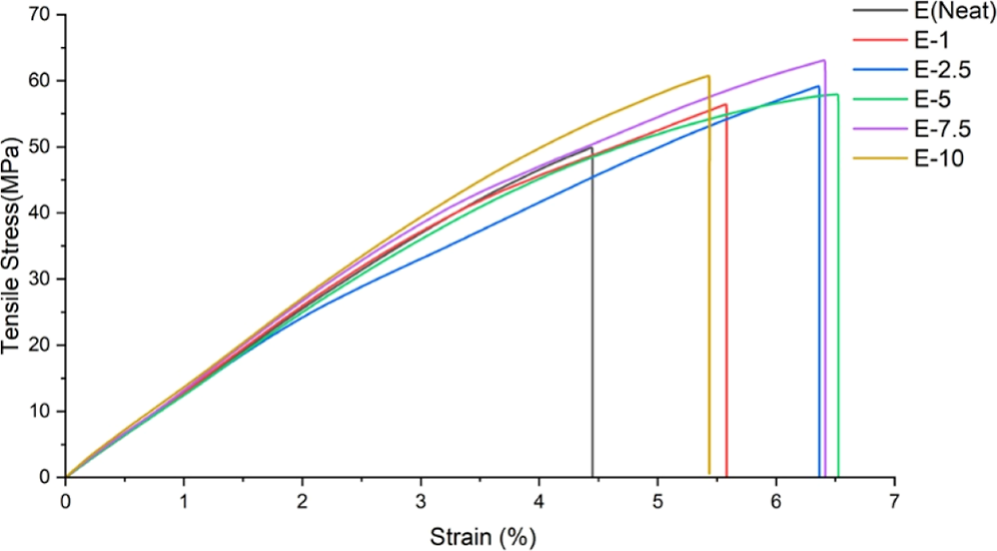
Tensile stress performance of tensile test specimens.

When the tensile test results of the study were examined, it was observed that the E-7.5 sample exhibited a tensile strength of 63.09 MPa, which is approximately 26% higher than that of the neat epoxy sample. Similar strengthening trends have been reported in natural fiber-reinforced epoxy systems containing lignocellulosic reinforcements such as jute, hemp, and flax.^50,51^ However, direct comparison between different studies remains limited due to variations in fiber morphology, processing techniques, resin systems, and filler loading levels. A comparable approach has been reported in epoxy composites containing wood dust particles, where optimum mechanical performance was observed at approximately 5–10 wt % filler loading; however, the maximum tensile strength reported in that study remained around 30 MPa, and therefore, a direct quantitative comparison with the present system remains limited due to differences in filler type and composite architecture.^52^ In the present work, the main objective is not to directly compete with existing composite systems but to demonstrate the reinforcing potential of tea stem waste as an agro-industrial byproduct while partially reducing the amount of epoxy matrix required.

### 3.3 Determination of Fracture Toughness Values

The fracture toughness of the epoxy matrix increased consistently with the addition of tea stem powder in the presence of 0.5 wt % BNNP. As shown in Figure 5, the neat epoxy exhibited an average fracture toughness of approximately 1.68–1.70 MPa·m^1/2^. When 1 wt % filler is introduced (E-1), the value rises to around 1.84–1.90 MPa·m^1/2^, indicating that the hybrid reinforcement begins to improve crack resistance even at low loading. At 2.5 wt % (E-2.5), fracture toughness reaches roughly 1.94–1.95 MPa·m^1/2^, representing a clear enhancement relative to both neat epoxy and E-1. The 5 wt % composite (E-5) maintains a similar level (1.90–1.95 MPa·m^1/2^), showing that the toughening effect remains stable without any notable deterioration.

**Figure 5:**
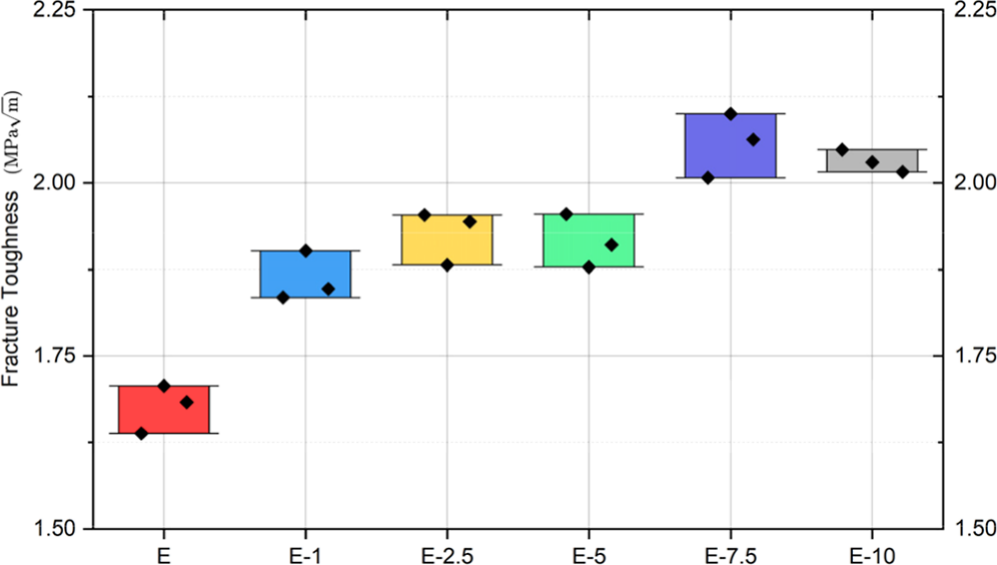
Fracture toughness (*K*_IC_) of the epoxy composites with the tea stem powder and 0.5 wt % BNNP.

At 7.5 wt % filler content (E-7.5), the fracture toughness reaches the highest value in the series, about 2.06–2.10 MPa·m^1/2^. This is a significant improvement. The toughness stays above 2.00 MPa·m^1/2^ even at 10 wt % (E-10), which shows that the better crack-resistance ability stays the same even with more filler. It is important to note that each composition was tested with three separate specimens. The plotted values show the average behavior of these replicates, and the error bars show the variation in each batch.

In a study on an epoxy matrix with bamboo fibers, fracture toughness tests with compact tension specimens showed that fracture toughness increased with increasing fiber length, 1.61 MPa m^1/2^ for 10 mm and 2.67 MPa m^1/2^ for 25 mm.^53^ Another study involving natural fiber knitted fabrics and SENB-type specimens made with epoxy shows fracture toughness values ranging from 2.57 MPa m^1/2^ to 3.64 MPa m^1/2^ were obtained with different knitted fabric types and densities.^54^ According to previous studies, the fracture toughness values obtained in terms of the ease of application and simple processing of fibers are considered appropriate.

### 3.4 Analysis of Fracture Surface Features of Epoxy Composites

The fracture morphology of the epoxy composites was examined by SEM to evaluate the influence of the filler concentration on crack propagation behavior. As shown in Figure 6a, the E-1 composite exhibits relatively smooth fracture features dominated by brittle epoxy fracture characteristics. Riverlike patterns and relatively flat regions can be observed, indicating that crack propagation mainly occurs through the epoxy matrix with limited microstructural disturbance. At this low filler content, the influence of the tea stem particles and BNNPs on the fracture path remained relatively limited.

**Figure 6:**
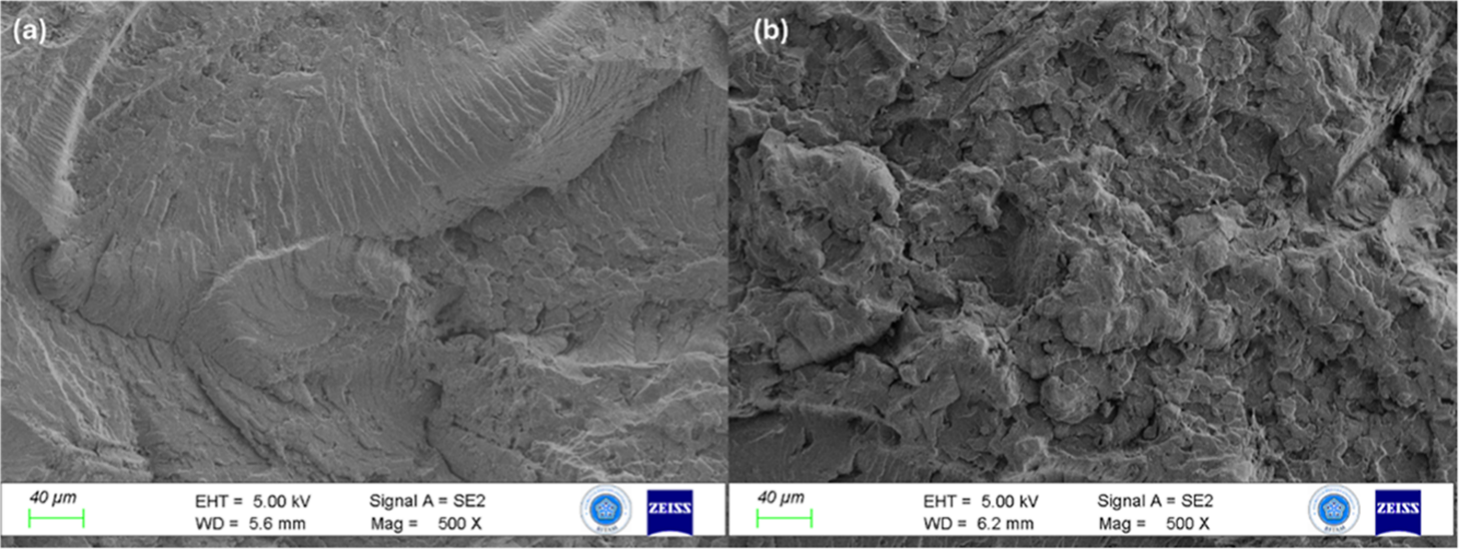
SEM images of fracture surfaces of epoxy composites: (a) E-1 composite and (b) E-10 composite.

In contrast, the fracture surface of the E-10 composite (Figure 6b) is significantly rougher and more heterogeneous. Numerous irregular features, microvoids, and particle-related structures are visible, suggesting that the higher filler content introduces multiple obstacles to crack propagation. These features indicate increased crack deflection and a more tortuous fracture path, which are commonly associated with enhanced energy dissipation during the fracture.

The high-magnification SEM images in Figure 7 correspond to the composite containing 10 wt % tea stem powder and 0.5 wt % boron nitride nanoplatelets (BNNPs) and reveal a heterogeneous fracture surface governed by locally competing toughening and damage mechanisms. The fracture surface is characterized by regions of matrix-dominated brittle cracking, particularly in particle-depleted epoxy-rich zones, where crack initiation occurs in the absence of an effective reinforcement. Several localized stress concentration zones were observed near the sharp morphological features and fractured lamellar structures, as highlighted in the magnified views. These regions are closely associated with agglomerated nanoplatelet clusters, which act as microstructural discontinuities and promote agglomerate-induced damage initiation rather than crack deflection. The presence of such agglomeration regions disrupts local load transfer and leads to premature crack initiation in the surrounding epoxy matrix.^55^

**Figure 7:**
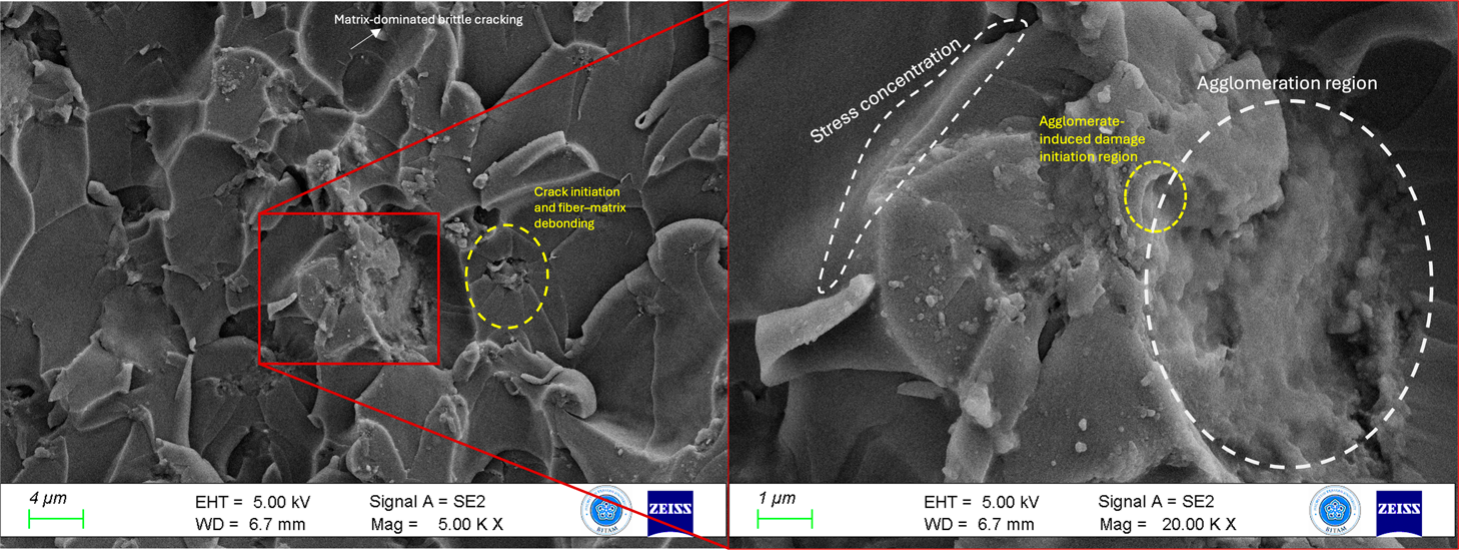
5000× and 20,000× magnified fracture regions of the E-10 sample (10% tea stem powder and 0.5% BNNP).

In contrast, adjacent areas exhibited particle–matrix debonding and microvoid formation, indicating localized energy-dissipation mechanisms associated with the lignocellulosic particulate reinforcement. However, the effectiveness of these mechanisms is limited by the nonuniform reinforcement distribution. Overall, the fracture morphology demonstrates that while hybrid reinforcement can promote crack deflection and energy absorption, excessive local agglomeration of BNNPs shifts the dominant fracture behavior toward stress-concentration-driven damage initiation and brittle matrix cracking.

### 3.5 FTIR Analysis of Tea Stem Powder, BNNP, and Epoxy Composites

The FTIR spectra of the tea stem powder, pristine boron nitride nanoplatelets (BNNPs), and representative epoxy composites are presented in Figure 8. The spectra were used to identify the characteristic functional groups of the lignocellulosic filler and nanofiller and to evaluate possible changes in the chemical structure of the epoxy matrix after hybrid reinforcement.

**Figure 8:**
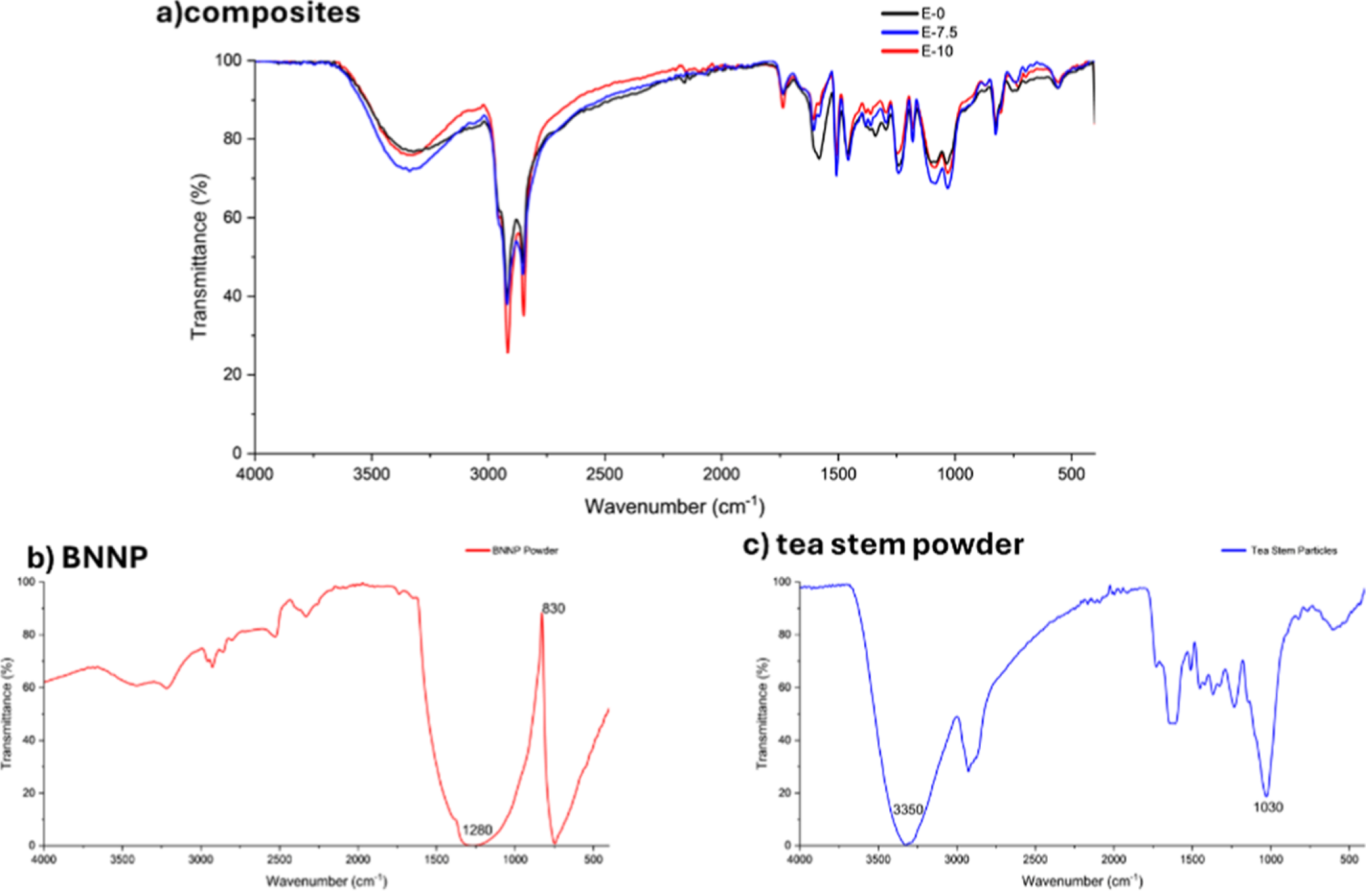
FTIR spectra of representative epoxy composites (E-7.5 and E-10), tea stem powder, and pristine boron nitride nanoplatelets (BNNPs).

The spectrum of the tea stem powder exhibits the typical absorption bands of lignocellulosic materials. A broad band centered around ∼3400 cm^–1^ is attributed to O–H stretching vibrations associated with hydroxyl groups in cellulose and hemicellulose. The peaks observed near 2920 cm^–1^ correspond to C–H stretching vibrations of aliphatic groups present in the lignocellulosic structure. In addition, the absorption bands in the 1000–1100 cm^–1^ region are mainly related to C–O and C–O–C stretching vibrations originating from cellulose and hemicellulose components. These characteristic bands confirm the typical chemical composition of untreated tea stem waste.

The spectrum of pristine BNNP shows the characteristic vibrational features of hexagonal boron nitride. A strong absorption band appears around ∼1360 cm^–1^, which corresponds to the in-plane B–N stretching vibration, while another prominent band is observed near ∼800 cm^–1^, attributed to the B–N–B out-of-plane bending vibration. These bands are consistent with previously reported FTIR spectra of hexagonal boron nitride nanoplatelets and confirm the chemical identity of the nanofiller used in this study.

The FTIR spectra of the epoxy composites exhibit the characteristic absorption bands of the cured epoxy network. The main peaks associated with the epoxy matrix remain largely preserved after the incorporation of tea stem powder and BNNPs. In particular, the broad absorption around ∼3400 cm^–1^, the aliphatic C–H stretching bands near 2920–2850 cm^–1^, and the C–O-related bands in the 1000–1200 cm^–1^ region remain clearly visible in the composite spectra. Only minor variations in peak intensity are observed with increasing tea stem content. The absence of significant peak shifts or the appearance of new absorption bands suggests that the incorporation of tea stem powder and BNNP does not introduce major changes in the chemical structure of the cured epoxy network. The hybrid fillers, therefore, appear to influence composite behavior primarily through physical reinforcement and interfacial interactions, rather than through the formation of new chemical bonds within the epoxy matrix.

### 3.6 DMA of Neat Epoxy and E-10 Composites

DMA measurements were conducted for neat epoxy (E-0) and the composite with the highest filler loading (E-10) in order to clearly evaluate the influence of hybrid reinforcement on viscoelastic behavior. The DMA results, presented in Figure 9, revealed a clear glass transition region for both the neat epoxy and the hybrid composites, characterized by a pronounced tan δ peak located at approximately 94–97 °C. This temperature range is in line with the expected glass transition behavior of postcured DGEBA-based epoxy systems. The addition of tea stem particles and boron nitride nanoplatelets (BNNPs) changes the viscoelastic behavior of the epoxy matrix to a limited extent, especially in the glassy region and transition region.

**Figure 9:**
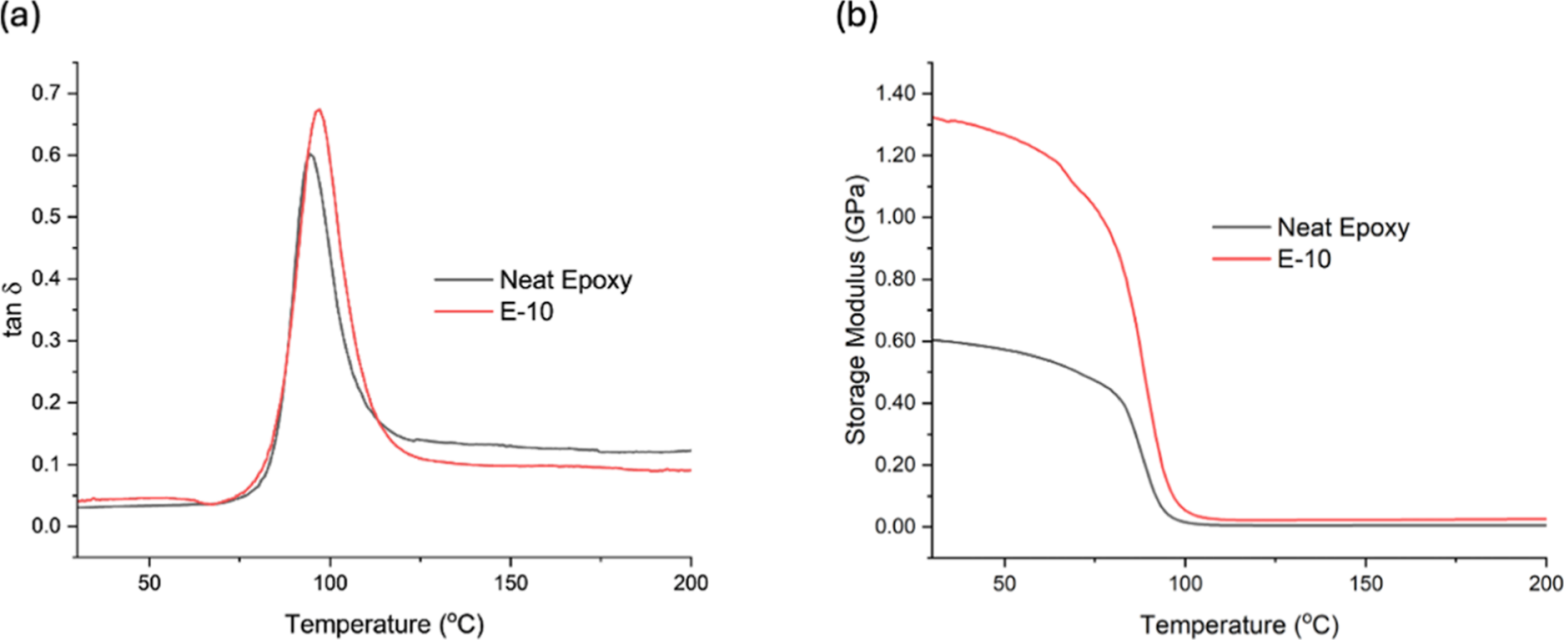
DMA results of E-0 and E-10 samples: (a) tan δ and (b) storage modulus comparison.

The storage modulus (*E*′) curves demonstrate that the hybrid composite exhibits significantly higher stiffness in the glassy region (0–80 °C) compared with neat epoxy. In particular, the E-10 formulation shows an *E*′ value exceeding 1.32 GPa, which is nearly twice that of the unfilled matrix. This increase can be attributed to the rigid nature of the lignocellulosic tea stem particles and the reinforcing effect of BNNPs. The fillers act as mechanically stiff inclusions that restrict the segmental mobility of the epoxy chains and improve load transfer within the composite. As a result, the composite structure becomes more resistant to deformation under oscillatory loading.

The loss modulus (*E*″) and tan δ responses provide additional insight into the relaxation behavior of the system. The E-10 composite exhibited a slightly higher tan δ peak than neat epoxy, indicating a modest increase in damping capability in the presence of tea stem particles and BNNPs. The peak width was evaluated using the full width at half-maximum (FWHM), and the peak sharpness was further expressed by the peak factor (tan δ_max_/FWHM). The main viscoelastic parameters obtained from the DMA curves, including *T*_g_ from the tan δ peak and representative storage modulus values in the glassy and post-*T*_g_ regions, are summarized in Table 2. Overall, the hybrid composite shows a slightly higher *T*_g_, a narrower relaxation interval, and improved storage modulus retention compared with neat epoxy. The narrower relaxation behavior may also be associated with the presence of filler phases that influence the interfacial polymer dynamics, as commonly observed in polymer nanocomposites.^56^

**Table 2:** Viscoelastic Parameters Obtained from DMA Curves

sample	*T*_g_ from tan δ (°C)	tan δ_max_	FWHM (°C)	peak factor (tan δ_max_/fwhm)	*E*′ at 30 °C (GPa)	*E*′ post-*T*_g_ (GPa)
E-0	94.33	0.600	15.39	0.039	0.60	0.011
E-10	97.10	0.674	10.04	0.067	1.32	0.026

The glass transition temperature (*T*_g_), estimated from the tan δ peak position, showed only a slight shift toward a higher temperature in the hybrid composite. This modest shift indicates that the fillers primarily influence the viscoelastic response through physical restriction of polymer chain mobility rather than through major changes in the curing chemistry of the epoxy network. Taken together, the *T*_g_ position, reduced fwhm, increased peak factor, and higher storage modulus indicate that the hybrid reinforcement modifies the relaxation behavior of the epoxy system while improving stiffness and maintaining thermal–mechanical stability.

### 3.7 Thermal Analysis (DSC and TGA) of the Samples

The thermal degradation behavior of the composites was investigated using TGA and DSC. As Figure 10 shows, the TGA curves show the main mass loss region (peak degradation temperature, *T*_p_) starting at approximately 280–320 °C for all samples, and this temperature range corresponds to the simultaneous degradation of both the epoxy matrix^57^ and the natural fibers. The fact that the fibers were not subjected to any surface modification caused variability from sample to sample, especially in the content of hemicellulose, pectin, wax, and minerals, resulting in significant differences in both the initial temperature and rate of degradation. This inherent heterogeneity was particularly evident in the amount of residue at high temperatures. When examining the TGA data at the final test temperature of 635 °C, it was observed that the results increased with the addition of tea stem powder and BNNP, reaching 6.95% for the pure epoxy. In the E-5 sample, this value is 9.58%, while for E-7.5 and E-10, it is 8.24% and 7.52%, respectively.

**Figure 10:**
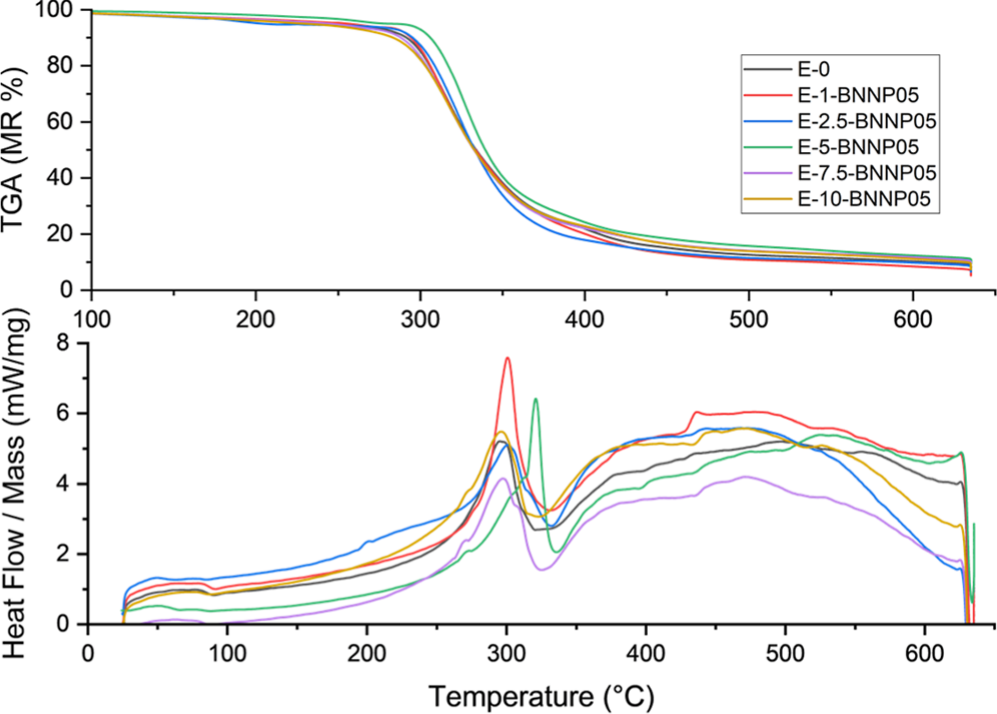
TGA and DSC thermograms of epoxy composites containing 0–10 wt % tea stem powder with 0.5 wt % BNNP.

The DSC results were consistent with the TGA data. The pronounced endothermic peak observed at approximately 300 °C represents the main thermal decomposition of the epoxy chains, followed by an exothermic region indicating a carbonization trend associated with the decomposition of the lignin-rich tea stem particles. The broad endothermic zone that reappears in the 400–500 °C range indicates a secondary degradation step of the epoxy matrix. The untreated surface of the tea stem particles caused the intensity of these thermal processes and their temperature locations to vary from sample to sample, so that both endothermic and exothermic signals showed marked differences.

In this study, no chemical treatment was applied to the tea stem powder obtained from tea stem waste. This was chosen to observe the organic-matter-derived variation in these large quantities of waste and facilitate its application. Although this resulted in a heterogeneous distribution in the thermal evaluation, the clear improvement observed compared to pure epoxy and the decomposition at higher temperatures are promising in terms of cost-effective applications for commercial use.

## 4 Conclusion

In this study, hybrid epoxy nanocomposites reinforced with tea stem waste powder and boron nitride nanoplatelets (BNNPs) were successfully fabricated and systematically characterized in terms of their hardness, tensile strength, fracture toughness, viscoelastic response, and thermal stability. The incorporation of lignocellulosic tea stem powder together with 0.5 wt % BNNP significantly improved the mechanical performance of the neat epoxy matrix. The Shore D hardness increased from 79.4 for neat epoxy to values exceeding 97 for all filled systems, with the highest hardness observed for the E-7.5 and E-10 formulations. Tensile testing showed a clear strengthening effect with increasing tea stem content, culminating in a maximum tensile strength of 63.09 MPa for the E-7.5 composite, while the E-10 composite retained a similarly high strength of 60.75 MPa. Fracture toughness was also enhanced across the series: *K*_IC_ increased from approximately 1.68–1.70 MPa m^1/2^ for neat epoxy to about 2.06–2.10 MPa m^1/2^ for E-7.5 and remained above 2.00 MPa m^1/2^ even at 10 wt % filler content, indicating robust crack-resistance in all hybrid systems.

The DMA further showed that the E-10 composite had a narrower tan δ peak (fwhm: 10.04 °C) than neat epoxy (15.39 °C), together with a higher peak factor, indicating a more localized α-relaxation behavior in the hybrid system. The composites displayed a sharp glass transition, which is consistent with that of postcured commercial DGEBA-based epoxy systems. In particular, the E-10 formulation showed a more pronounced and slightly right-shifted tan δ peak accompanied by a strong increase in storage modulus at high temperatures, with values exceeding 1.20 GPa—almost twice that of the pure matrix, owing to the rigid filler effect. This is attributed to the limited chain mobility and enhanced energy dissipation due to the coupled effect of the tea stem particles and BNNPs. From the general viscoelastic behavior, it can be concluded that the hybrid composites still possess a thermally stable cross-linking network but with higher stiffness and loss tangent than the unmodified epoxy.

The TGA and DSC results are consistent with the mechanical and DMA observations. For all filled materials, the main degradation occurred in the same temperature range (ca. 280–320 °C), corresponding to the simultaneous decomposition of the epoxy matrix and lignocellulosic components. By incorporating tea stem powder and BNNP, the residual char at 635 °C increased from 6.95% for neat epoxy to 9.58% for E-5 and E-7.5 and E-10 displayed 8.24% and 7.52% char, respectively, indicating a higher thermal stability and a thermally stable carbonaceous structure. The DSC diagrams exhibited a strong endothermic signal at approximately 300 °C and exothermic effects associated with carbonization and residual degradation processes. The intensity and position of these thermal events varied due to the inherent heterogeneity of the untreated tea stem particles.

When all of the results are considered, the composites containing 7.5 and 10 wt % tea stem powder, along with 0.5 wt % BNNP, both showed improved mechanical and thermal performance compared with neat epoxy, with higher hardness, strength, fracture toughness, stiffness, and char residue. However, the differences between the E-7.5 and E-10 formulations were relatively small for most of the measured properties. In many cases, E-7.5 provided the highest or near-highest tensile strength and fracture toughness, despite having a slightly lower filler content. Considering the mechanical and thermal results together, E-7.5 can therefore be regarded as the optimum formulation within the investigated range, providing a balanced combination of performance improvement, material efficiency, and processability.

The hybrid fillers introduce combined reinforcement mechanisms that contribute to the enhanced mechanical performance. The lignocellulosic tea stem particles mainly contribute to bulk stiffening and crack-path modification, while the BNNPs contribute to local stress redistribution and stiffness improvement. In line with the FTIR results, these improvements are mainly attributed to physical reinforcement and interfacial effects rather than to the formation of new chemical bonds in the epoxy network. To amplify the differences among the compositions, future work will address the adjustments of tea stem reinforcement. Improvements could be obtained by introducing surface treatments for the lignocellulosic tea stem particles, such as functionalization or coupling agents, along with better control of particle size. These applications may improve particle–matrix adhesion and lead to a more uniform composite structure. These changes could lead to higher increases in the tensile strength, fracture toughness, thermal stability, and viscoelastic behavior, and these modifications work well to clearly differentiate the performance between 7.5 and 10 wt % systems. Further investigations, such as of the BNNP loadings that are alternative and thermal conductivity and long-term durability, might present a much broader vision about the potential for structural and thermally challenging applications of tea stem/BNNP composites along with epoxy.

## Supplementary Material



## Notes

Additional figures showing mold designs, specimen preparation, and the experimental workflow (PDF)

## References

[1] Aziz T., Haq F., Farid A., Cheng L., Chuah L. F., Bokhari A., Mubashir M., Tang D. Y. Y., Show P. L. (2024). The Epoxy Resin
System: Function and Role of Curing Agents. Carbon Lett..

[2] Chen B., Zhang Q., Lu M., Meng H., Qu Z., Xu C., Jiao E. (2021). Synthesis
of a Novel lignin-based Epoxy Resin Curing
Agent and Study of Cure Kinetics, Thermal, and Mechanical Properties. J. Appl. Polym. Sci..

[3] Czaderski C., Martinelli E., Michels J., Motavalli M. (2012). Effect of
Curing Conditions on Strength Development in an Epoxy Resin for Structural
Strengthening. Composites, Part B.

[4] Kumar S., Krishnan S., Mohanty S., Nayak S. K. (2018). Synthesis and Characterization
of Petroleum and Biobased Epoxy Resins: A Review. Polym. Int..

[5] Pethrick R. A. (2015). Design
and Ageing of Adhesives for Structural Adhesive Bonding – A
Review. Proc. Inst. Mech. Eng., Part L.

[6] Wang J., Li M., Li Q., Chen J., Huang C., Li Y., Feng X. (2025). Enhancing
Thermal Conductive Properties With Liquid Metal-Assisted
Epoxy Resin Composites. Polym. Compos..

[7] Xu F., Cui Y., Bao D., Lin D., Yuan S., Wang X., Wang H., Sun Y. (2020). A 3D Interconnected Cu Network Supported
by Carbon Felt Skeleton for Highly Thermally Conductive Epoxy Composites. Chem. Eng. J..

[8] Choi J. R., Yu S., Jung H., Hwang S. K., Kim R. H., Cho S. H., Bae I., Hong S. M., Koo C. M., Park C. (2015). Self-Assembled Block
Copolymer Micelles with Silver-Carbon Nanotube Hybrid Fillers for
High Performance Thermal Conduction. Nanoscale.

[9] Yan F., Liu L., Li M., Zhang M., Xiao L., Ao Y. (2018). Preparation
of Carbon Nanotube/Copper/Carbon Fiber Hierarchical Composites by
Electrophoretic Deposition for Enhanced Thermal Conductivity and Interfacial
Properties. J. Mater. Sci..

[10] Hamdi L., Asma B., Ali B. (2024). Tensile Mechanical
Performance of
Natural/Natural Fiber Reinforced Hybrid Bio-Composite Materials –
A Statistical Approach. J. Ind. Text..

[11] Akhil U. V., Radhika N., Saleh B., Aravind Krishna S., Noble N., Rajeshkumar L. (2023). A Comprehensive
Review on Plant-based
Natural Fiber Reinforced Polymer Composites: Fabrication, Properties,
and Applications. Polym. Compos..

[12] Sharma N., Allardyce B. J., Rajkhowa R., Agrawal R. (2023). Rice Straw-Derived
Cellulose: A Comparative Study of Various Pre-Treatment Technologies
and Its Conversion to Nanofibres. Sci. Rep..

[13] Saba N., Paridah M. T., Abdan K., Ibrahim N. A. (2016). Effect of Oil Palm
Nano Filler on Mechanical and Morphological Properties of Kenaf Reinforced
Epoxy Composites. Constr. Build. Mater..

[14] Alves C., Ferrão P. M. C., Silva A. J., Reis L. G., Freitas M., Rodrigues L. B., Alves D. E. (2010). Ecodesign of Automotive Components
Making Use of Natural Jute Fiber Composites. J. Cleaner Prod..

[15] Mousavi-Avval S. H., Sahoo K., Nepal P., Runge T., Bergman R. (2023). Environmental
Impacts and Techno-Economic Assessments of Biobased Products: A Review. Renewable Sustainable Energy Rev..

[16] Khan T., Hameed Sultan M. T. B., Ariffin A. H. (2018). The Challenges of Natural Fiber in
Manufacturing, Material Selection, and Technology Application: A Review. J. Reinf. Plast. Compos..

[17] Faria D. L., Mendes L. M., Junior J. B. G. (2023). Effect of Surface
Treatment on the
Technological Properties of Coconut Fiber–Reinforced Plant
Polyurethane Composites. Environ. Sci. Pollut.
Res..

[18] Karthik K., Rajamanikkam R. K., Venkatesan E. P., Bishwakarma S., Krishnaiah R., Saleel C. A., Soudagar M. E. M., Kalam M. A., Ali M. M., Bashir M. N. (2024). State of the Art: Natural Fibre-Reinforced
Composites in Advanced Development and Their Physical/Chemical/Mechanical
Properties. Chin. J. Anal. Chem..

[19] Ashik K. P., Sharma R. S. (2015). A Review on Mechanical
Properties of Natural Fiber
Reinforced Hybrid Polymer Composites. JMMCE.

[20] Zhao F., Guo W., Liu X., Zhao J., Feng T. (2024). Injection Molded Lightweight
Composites from Tea-Stem Fiber and Polypropylene: Effect of Fiber
Loading on Forming Properties and Cell Structure. Ind. Crops Prod..

[21] Efe Ş. (2025). Industrial
Tea Waste and Energy Potential. J. Energy Trends.

[22] Kaya H., Ekrem M., Uslugil Y. (2025). Tea Stem Waste
Powders for Epoxy-Based
Composites: Processing, Mechanical Properties, and Fracture Behavior. El-Cezeri J. Sci. Eng..

[23] Borah N., Karak N. (2023). Green Composites of Bio-Based Epoxy and Waste Tea Fiber as Environmentally
Friendly Structural Materials. J. Macromol.
Sci., Part A:Pure Appl. Chem..

[24] Balaji D., Kumar P. S., Bhuvaneshwari V., Rajeshkumar L., Singh M. K., Sanjay M. R., Siengchin S. (2025). A Review on
Effect of Nanoparticle Addition on Thermal Behavior of Natural Fiber–Reinforced
Composites. Heliyon.

[25] Sathish
Kumar R., Vandhana Devi V., Nivedhitha D. M., Srish Satya S., Visakan S., Bharath Sharma S., Sathish Kumar S. B. (2026). Effect of Nanoparticles in Natural Fiber Reinforced
Polymer Composites. Mater. Today: Proc..

[26] Bahtiyar G., Ekrem M., Ünal B., Ak S. (2023). Mechanical Properties
and Damage Behaviours of Polyurethane Composites Reinforced With BNNP
and MWCNT Hybrid Nanoparticles. J. Elastomers
Plast..

[27] Ekrem M., Düzcükoğlu H., Ali Şenyurt M., Sinan Şahin Ö., Avcı A. (2018). Friction and
Wear Performance of Epoxy Resin Reinforced With Boron Nitride Nanoplatelets. J. Tribol..

[28] Demircan G., Kisa M., Ozen M., Aktas B. (2020). Surface-Modified Alumina
Nanoparticles-Filled Aramid Fiber-Reinforced Epoxy Nanocomposites:
Preparation and Mechanical Properties. Iran.
Polym. J..

[29] Mishra T., Mandal P., Rout A. K., Sahoo D. (2022). A State-of-the-Art
Review on Potential Applications of Natural Fiber-Reinforced Polymer
Composite Filled with Inorganic Nanoparticle. Compos., Part C: Open Access.

[30] Dejene B. K. (2024). Exploring
the Potential of ZnO Nanoparticle-Treated Fibers in Advancing Natural
Fiber Reinforced Composites: A Review. J. Nat.
Fibers.

[31] Rahman A., Madadi M., Ma J., Zhang P. (2025). Overcoming Conductivity–Stretchability
Trade-Off in Soft Conductive Composites through Liquid Metal Junctions. ACS Appl. Mater. Interfaces.

[32] Zhang W., Wu Z.-Y., Jiang H.-L., Yu S.-H. (2014). Nanowire-Directed
Templating Synthesis of Metal–Organic Framework Nanofibers
and Their Derived Porous Doped Carbon Nanofibers for Enhanced Electrocatalysis. J. Am. Chem. Soc..

[33] Madadi M., Zhang P. (2024). Finite-Size Effect on the Percolation and Electromechanical Behaviors
of Liquid Metal Particulate Composites. Soft
Matter.

[34] Dhamodharan D., Dhinakaran V., Byun H.-S. (2022). MXenes: An Emerging 2D Material. Carbon.

[35] Huang J., Zhou J., Liu M. (2022). Interphase
in Polymer Nanocomposites. JACS Au.

[36] Öztürkmen M. B., Öz Y., Dilsiz N. (2023). Physical and mechanical properties
of graphene and h-Boron nitride reinforced hybrid aerospace grade
epoxy nanocomposites. J. Appl. Polym. Sci..

[37] Gonçalves F. A.
M. M., Santos M., Cernadas T., Alves P., Ferreira P. (2022). Influence
of Fillers on Epoxy Resins Properties: A Review. J. Mater. Sci..

[38] George J. S., Vijayan P P., Ponçot M., Vahabi H., Maria H. J., Thomas S. (2024). Insights into the Synergistic
Effect of Graphene Oxide/Silica
Hybrid Nanofiller for Advancing the Properties of Epoxy Resin. ACS Appl. Polym. Mater..

[39] Sharker S. Md. (2019). Hexagonal
Boron Nitrides (White Graphene): A Promising Method for Cancer Drug
Delivery. IJN.

[40] Sukur E., Kaybal H., Ulus H., Avci A. (2023). Tribological Behavior
of Epoxy Nanocomposites under Corrosive Environment: Effect of High-Performance
Boron Nitride Nanoplatelet. Sigma J. Eng. Nat.
Sci..

[41] Hyun W., de Moraes A., Lim J., Downing J., Park K., Tan M., Hersam M. (2019). High-Modulus
Hexagonal Boron Nitride Nanoplatelet Gel
Electrolytes for Solid-State Rechargeable Lithium-Ion Batteries. ACS Nano.

[42] Chaurasia A., Parashar A., Mulik R. S. (2020). Effect of Hexagonal Boron Nitride
Nanoplatelet on Crystal Nucleation, Mechanical Behavior, and Thermal
Stability of High-Density Polyethylene-Based Nanocomposites. Macromol. Mater. Eng..

[43] Tasgin Y., Demircan G., Kandemir S., Açikgöz A. (2024). Mechanical,
Wear and Thermal Properties of Natural Fiber-Reinforced Epoxy Composite:
Cotton, Sisal, Coir and Wool Fibers. J. Mater.
Sci..

[44] Kumar S., Patel V. K., Mer K. K. S., Gangil B., Singh T., Fekete G. (2021). Himalayan Natural Fiber-Reinforced Epoxy Composites:
Effect of *Grewia Optiva/Bauhinia Vahlii* Fibers on
Physico-Mechanical and Dry Sliding Wear Behavior. J. Nat. Fibers.

[45] Jamshaid H., Mishra R., Basra S., Rajput A. W., Hassan T., Petru M., Choteborsky R., Muller M. (2022). Lignocellulosic Natural
Fiber Reinforced Bisphenol F Epoxy Based Bio-Composites: Characterization
of Mechanical Electrical Performance. J. Nat.
Fibers.

[46] Mohan T. P., Kanny K. (2019). Compressive Characteristics of Unmodified and Nanoclay Treated Banana
Fiber Reinforced Epoxy Composite Cylinders. Composites, Part B.

[47] Dhanasekar K., Krishnan A. M., Kaliyaperumal G., De Poures M. V., Chandramohan P., Parthipan N., Priya C. B., Venkatesh R., Negash K. (2023). Influences of Nanosilica
Particles on Density, Mechanical,
and Tribological Properties of Sisal/Hemp Hybrid Nanocomposite. Adv. Polym. Technol..

[48] Thakur A., Purohit R., Rana R. S., Bandhu D. (2018). Characterization and
Evaluation of Mechanical Behavior of Epoxy-CNT-Bamboo Matrix Hybrid
Composites. Mater. Today: Proc..

[49] Koyunbakan M., Uslugil Y., Ekrem M., Eser U. ¨. (2025). Investigation
of the Mechanical Properties of Aramid Fiber-Reinforced Hybrid Nanocomposites
with BNNP-Enhanced Epoxy Matrix. Compos. Interfaces.

[50] Sienkiewicz N., Dominic M., Parameswaranpillai J. (2022). Natural Fillers
as Potential Modifying
Agents for Epoxy Composition: A Review. Polymers.

[51] Mittal V., Saini R., Sinha S. (2016). Natural Fiber-Mediated
Epoxy Composites
– A Review. Composites, Part B.

[52] Kumar R., Kumar K., Sahoo P., Bhowmik S. (2014). Study of Mechanical
Properties of Wood Dust Reinforced Epoxy Composite. Procedia Mater. Sci..

[53] Khan Z., Yousif B. F., Islam M. (2017). Fracture Behaviour
of Bamboo Fiber
Reinforced Epoxy Composites. Composites, Part
B.

[54] Tiber B., Balcıoğlu H. E. (2019). Flexural
and Fracture Behavior of
Natural Fiber Knitted Fabric Reinforced Composites. Polym. Compos..

[55] Rao Y.-N., Dai H.-L., Dai T., Yang Y. (2017). Linkages among Fiber
Content, Porosity and Local Aggregation in Fiber-Reinforced Composites,
and Their Effect on Effective Properties. J.
Mater. Sci..

[56] Susan
George J., Vijayan P P., Ponçot M., Keloth Paduvilan J., Thomas S. (2024). Viscoelastic and Rheokinetic Behaviour
of Cellulose Nanofiber/ Cloisite 30B Hybrid Nanofiller Reinforced
Epoxy Nanocomposites. Chem. Eng. J..

[57] Gültekin K., Uğuz G., Topcu Y., Özel A. (2021). Structural,
Thermal, and Mechanical Properties of Silanized Boron Carbide Doped
Epoxy Nanocomposites. J. Appl. Polym. Sci..

