# Supplementary material for: Mechanical, Viscoelastic, and Fracture Characteristics of Tea Waste and Boron Nitride Nanoplatelet-Reinforced Epoxy Composites
Source: ACS Omega. 2026 May 14;11(21):31220–30. doi: 10.1021/acsomega.6c00934 (PMC13234891; doi:10.1021/acsomega.6c00934)
Supplement: Supplementary file 1 [file ao6c00934_si_001.pdf]

Supporting Information for Publication

Mechanical, viscoelastic and fracture characteristics of tea waste and boron nitride nanoplatelet reinforced epoxy composites

Yasin Uslugil [1,] , Hüseyin Kaya [2] and Mürsel Ekrem [*3]

[1] Department of Mechatronics Engineering, Faculty of Engineering and Nature Sciences, KTO Karatay University, Konya, 42010, Türkiye

[2] Department of Machine and Metal Technologies, Technical Sciences Vocational School, Karamanoğlu Mehmetbey University, Karaman, 70100, Türkiye

[3] Department of Mechanical Engineering, Necmettin Erbakan University, Konya, 42005, Türkiye

* Correspondence: mekrem@erbakan.edu.tr

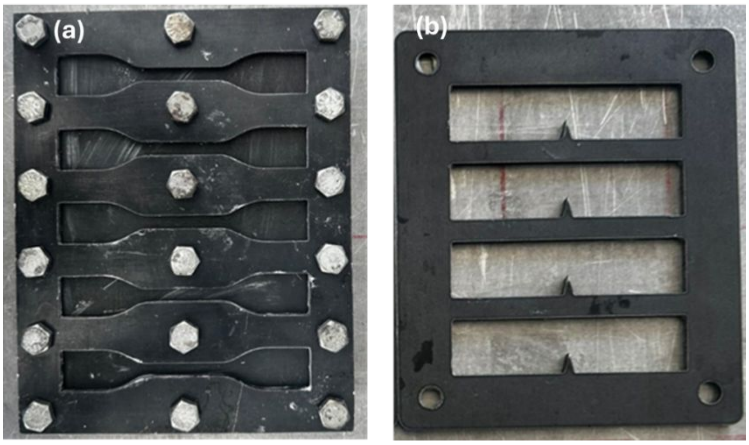

Figure S1. Steel molds for tensile (a) and fracture (b) tests according to ASTM D638 and ASTM D5045.

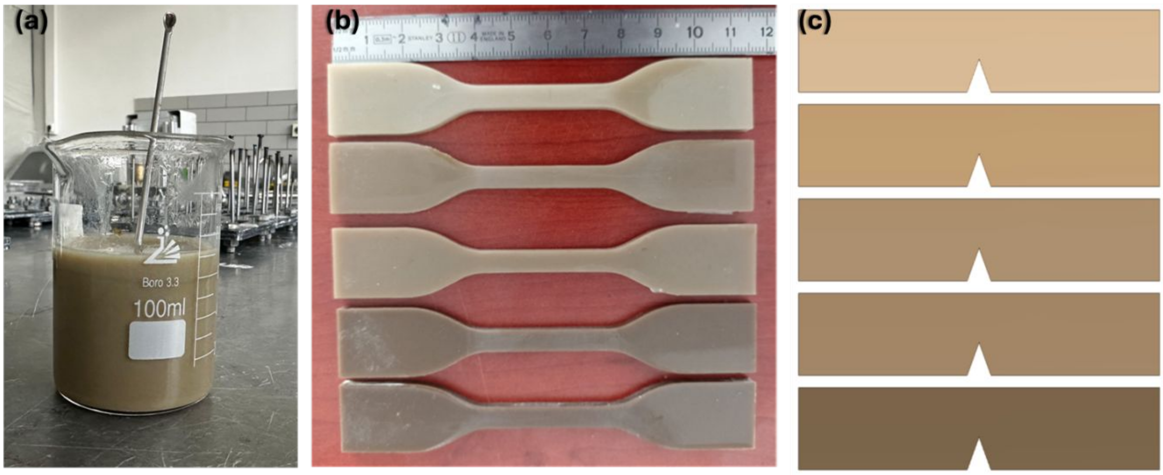

Figure S2. Composite specimen preparation and geometry: (a) mixture of epoxy, hardener, tea stem powder, and BNNP during homogenization; (b) tensile test specimens fabricated in accordance with ASTM D638 Type IV; and (c) schematic representation of fracture toughness specimens with a single-edge notch configuration (ASTM D5045).

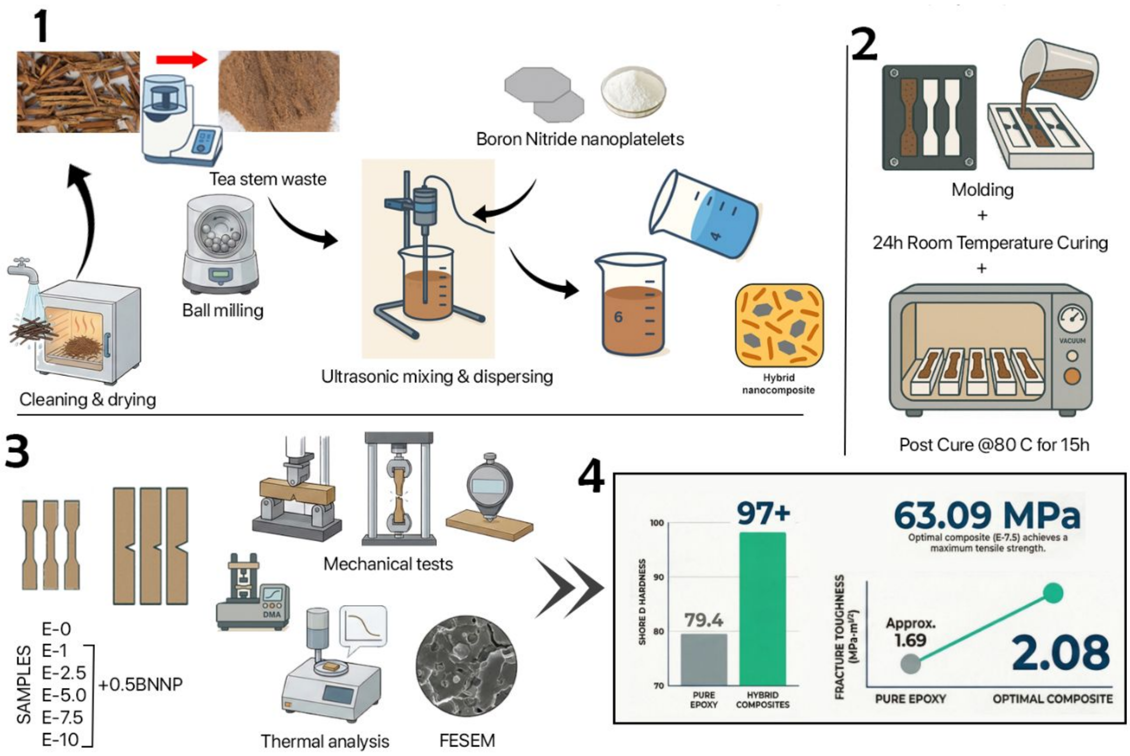

Figure S3. Graphical summary of the preparation, fabrication and characterization workflow of the tea stem waste/BNNP reinforced epoxy composites.
